# Prevalence and determinant factors of depression and anxiety in people with chronic kidney disease: a Moroccan cross-sectional study

**DOI:** 10.11604/pamj.2024.48.15.42881

**Published:** 2024-05-20

**Authors:** Amina Chrifi Alaoui, Mohammed Omari, Noura Quarmich, Omar Kouiri, Basmat Amal Chouhani, Mohammed Berraho, Nabil Tachfouti, Tarik Sqalli Houssaini, Samira El Fakir

**Affiliations:** 1 Department of Epidemiology, Clinical Research and Community Health, Faculty of Medicine and Pharmacy, Sidi Mohamed Ben Abdellah University, Fez, Morocco,; 2 Department of Nephrology, Hassan II University Hospital, Fez, Morocco,; 3 Laboratory of Epidemiology and Health Sciences´ Researches (ERESS), Faculty of Medicine and Pharmacy, Sidi Mohamed Ben Abdallah University, Fez, Morocco

**Keywords:** Depression, anxiety, prevalence, chronic kidney disease

## Abstract

**Introduction:**

the chronic kidney disease (CKD), is associated with various psychiatric conditions and poorer quality of life. The main objective of this study is to estimate the prevalence of depression and anxiety among CKD patients and to assess their determinant factors.

**Methods:**

this is a cross-sectional study in a Moroccan university hospital. Patients aged ≥18 years and followed for more than one year for a stage 3 to 5 CKD, not under renal replacement therapy (RRT) were included. The data were collected using a questionnaire and the hospital anxiety and depression scale (HADS). The association between depression and anxiety sub-scores and the estimated glomerular filtration rate (eGFR) was assessed using simple and multiple linear regression.

**Results:**

eighty-three patients (mean age 61.7 ± 14.1 years, and 61.4% were women), were included. Regarding the stages of CKD, 10.7% were in stage 3, 52.2% in stage 4, and 25% in stage 5 of CKD. The median of the depression sub-score was 5.00 (IQR (2.00; 10.0)). The median of the depression sub-score was 5.00 (IQR (2.00; 10.09)), and the median of the anxiety sub-score was 6.00 (IQR (4.00; 9.00)). It also shows that 22.0% of patients had depression (stage 4 = 23.8%, stage 5 = 36.8%) and 22.2% had anxiety (stage 4 = 26.2%, stage 5 = 33.3%). Both depression and anxiety scores were significantly associated with the eGFR before (p<0.001, p= 0.001 respectively) and after adjustment (p=0.001, p=0.002 respectively).

**Conclusion:**

according to this study depression and anxiety are strongly related to eGFR.

## Introduction

The chronic kidney disease (CKD), defined as abnormalities of the kidney´s structure or function, present for more than 3 months with implications for health [1], is a major health problem that negatively affects patients´ social, financial, and psychological well-being [2,3]. Indeed, patients affected with this chronic illness are highly susceptible to emotional problems because of the chronic stress related to disease burden, dietary restrictions, functional limitations, associated chronic diseases, adverse effects of medication, changes in self-perception, and fear of death [4].

In 2017, the Global Burden Disease (GBD) kidney disease collaboration study [5], showed that the number of cases of CKD worldwide (all stages combined) is 697.5 million (95% uncertainty interval (UI): 649.2 million - 752.0 million). The global prevalence of CKD has been estimated at 9.1% (95% UI: 8.5% - 9.8), with stages 1-2 accounting for 5.0% (95% UI: 4.5% - 5.5%), stage 3 representing 3.9% (95% UI: 3.5% - 4.3%), stage 4 representing 0.16% (95% UI: 0.13% - 0.19 %), and stage 5 representing 0.07% (95% UI: 0.06% - 0.08%) [5].

In the systematic review realized by Palmer *et al*. [6], 22.8% (95% confidence interval (CI): 18.6%-27.6%)) of patients undergoing dialysis and 21.4% (95% CI: 11.1% - 37.2%) of patients with pre-dialysis CKD suffered from depression disorder diagnosed by a structured clinical interview. However, if the diagnosis of depression is made using a self- or clinician-administered rating scale, this prevalence increased to 39.3% (95% CI, (36.8 - 42.0)) and 26.5% (95% CI, (18.5 - 36.5)) respectively.

In 2020, the GBD kidney disease collaboration estimated that the incidence of CKD in Morocco was 11.4/100,000 inhabitants of which 40% were diabetics [7] and that the current number of patients affected with CKD is 3,289,444 (95% UI: 3,046,873 - 3,568,865) [5], which is equivalent to a prevalence of 9.2%.

World Health Organization (WHO) reports on the Moroccan population's mental health, showed that 1.48 million Moroccans suffered from a depressive disorder which represents 4.5% of the total population, and 1.47 million one suffered from anxiety which also accounted for 4.5% of the population [8]. In the local literature, to the best of our knowledge, the prevalence of depression and anxiety disorders were studied only in chronic hemodialysis patients but not in people with CKD before the start of renal replacement therapy [9,10].

The main objective of this study is to estimate the prevalence of depression and anxiety among CKD patients and to assess their association with sociodemographic and clinical factors.

## Methods

**Study design:** this is a cross-sectional study, carried out in the outpatient visits and day care hospitals, in the Department of Nephrology and Dialysis affiliated to a university hospital in Morocco, between October 2019 and October 2020.

**Study population:** patients aged 18 years old or older, followed for more than a year for a stage 3 to 5 of CKD defined as follows: stage 3 = estimated filtration rate (eGFR) between 59 and 30 ml/min/1.73 m^2^, stage 4 = eGFR between 15 and 29 ml/min/1.73 m^2^, and stage 5= eGFR < 15 ml/min/1.73 m^2^, not dialyzed nor transplanted and agreeing to participate to the study were eligible for enrollment. The minimum sample size was estimated at 66 using the following formula [11]:


$$N=\frac{\left(Z^{2}*P\left(1-P\right)\right)}{d^{2}}$$

Where Z is the statistic for a level of confidence (1.96 for 95% confidence level); P = expected prevalence of anxiety or depression (4.5% [8]), and d = precision (0.05).

**Data sources and variables:** the sociodemographic and clinical data were collected from medical records using a questionnaire and the depression and anxiety data were collected by interview (face-to-face or by phone) using the hospital anxiety and depression scale (HADS) [12].

The sociodemographic section aimed to collect the data about age, gender, marital status, number of dependents and children, life condition, educational level, working status, monthly income, and health insurance. The clinical section collected data about the comorbidities, the initial nephropathy and its duration, the latest results of some laboratory tests (hemoglobin, iron, ferritin, creatinine, and urea), high blood pressure (HBP) treatment (angiotensin II receptor inhibitors (ARI II), ACE, calcium channels inhibitors (CI), diuretics), and supplementation medication (calcium, vitamin D, iron and erythropoietin).

As for depression and anxiety data, it was collected using HADS, previously validated in the Moroccan Arabic dialect [13]. The HADS was developed in 1983 by Zigmond and Snaith, and aims to assess the depression and anxiety symptoms in medical services patients, who usually suffer from organic problems. This tool helps look for anxio-depressive symptomatology and assess its severity, without distinguishing the different types of depression nor anxiety [12]. It combines two sub-scales: 1) Depression: 7 items (2, 4, 6, 8, 10, 12, and 14) to assess the depression, one for the dysphoria, one for the slow-down, and five items for the anhedonia dimension; 2) anxiety : 7 items (1, 3, 5, 7, 9, 11, and 13) to assess anxiety from the present state examination [14] and clinical anxiety scale [15].

**Statistical analysis:** each item had 4 response´s modalities coded from 0 to 3 or 3 to 0. The subscale scores were calculated in the same way and varied between 0 and 21, the best score being the lowest. The threshold for the sub-scores indicating the existence or not of depression or anxiety is 10 (0 to 10 = no depression or anxiety, and 11 to 21 = recognized anxiety or depressive disorders).

The sociodemographic and clinical characteristics and HADS scores were presented as numbers and percentages for qualitative variables and mean ± standard deviation (SD) or median and interquartile rate [IQR] for the quantitative variables according to their distribution tested using the Shapiro-Wilk test.

The HADS sub-scores were compared between the CKD stages using the Kruskal-Wallis non-parametric test, and their association with the eGFR was assessed using a Spearman correlation. Then a simple linear regression was used to assess the association between depression/anxiety and sociodemographic and clinical factors. Finally, the association between HADS scores and eGFR was reassessed after adjustment on some confounding factors using a stepwise backward multiple linear regression. All tests were two-tailed, and the threshold of significance was p <0.05. The statistical analysis was performed using the packages “car”, “prettyR”, and “compare groups”, of the version 3.6.1 of R software. All necessary authorizations for this study were obtained including the ethics committee approval.

## Results

**Population characteristics:** overall, 88 patients were approached, but only 83 patients (mean age 61.7 ± 14.1 years and 61.4% were women) accepted to participate in the study. Regarding the stages of CKD, 10.7% of them were in stage 3, 52.2% in stage 4, and 25% in stage 5 of CKD. Concerning marital status and living conditions. The majority were married (76.5%), 85% lived as a couple, and 10% lived with their family. As for the level of education, more than half of the patients were illiterate or attended Koranic school (58.7%). The majority of patients were unemployed (73.8%), and 68.8% had a monthly income ≤ 2000 MAD. Concerning health insurance 20.7% were affiliated with mandatory health insurance.

Regarding comorbidities, 30.9% were diabetic, 67.9% had high blood pressure (HBP), and 26.6% had a history of cardiovascular diseases. As for the initial nephropathy, 22.9% had diabetic nephropathy, 24.1% had glomerulonephritis, 14.5% had hypertensive nephropathy, 10.8% had vascular nephropathy, 3, 61% had tubulointerstitial nephropathy. The median eGFR was estimated at 22.0 ml/min/1.73 m^2^(IQR: 15.0; 31.0), and the median duration of CKD was 48 months (IQR: 26; 81).

The median serum urea level was 0.84 g/l (IQR: 0.62;1.27), the median serum creatinine level was 24.0 mg/l (IQR: 18.0; 37.0), the mean of hemoglobin was 11, 3 ± 2.06 g/dl, the median serum iron and ferritin levels were 0.69 μg/l (IQR: 0.43; 0.84) and 75.0 ng/ml (IQR: 37.0; 188) respectively. As for medication, 28.9% were on angiotensin II receptor inhibitors (ARI II), 26.5% were on ACE inhibitors, and 37.3% were on CI, 15.7% were taking calcium, 18.2% were taking vitamin D; 27.7% were taking iron, and 7.23% were on erythropoietin. Table 1 displays the description of sociodemographic and clinical characteristics according to the CKD stages.

**Table 1:** description of sociodemographic and clinical characteristics according to chronic kidney disease (CKD) stage

Sociodemographic and clinical characteristics	Stade 3 (N=21)	Stade 4 (N=43)	Stade 5 (N=19)
Age (years) (mean ± SD)	60.3 ± 15.2	60.8 ± 14.3	65.5 ± 12.4
Gender (women) (n, %)	10 (47.6%)	28 (65.1%)	13 (68.4%)
Marital status (n, %)			
Single / widow(er)/ divorced	3 (14,28%)	9 (21, 14%)	2 (38, 86%)
Married	18 (85.7%)	33 (78.6%)	11 (61.1%)
Life condition (n, %)			
Alone	1 (4.76%)	2 (4.76%)	1 (5.88%)
As a couple	17 (81.0%)	36 (85.7%)	15 (88.2%)
As a family	3 (14.3%)	4 (9.52%)	1 (5.88%)
*Niveau d'éducation* (n, %)			
*Analphabète /coranique*	10 (47.6%)	24 (57.1%)	13 (76.4%)
Elementary /university	11 (52.4%)	18 (42.9%)	4 (23.6%)
Working status (n, %)			
Active	3 (14.3%)	8 (19.0%)	2 (11.8%)
Retired	4 (19.0%)	4 (9.52%)	0 (0.00%)
Unemployed	14 (66.7%)	30 (71.4%)	15 (88.2%)
Monthly income (n, %)			
≤200 US$	18 (85.7%)	24 (57.1%)	13 (76.5%)
>200 US$	3 (14.28%)	9 (21.42%)	0 (0.00%)
Health insurance (n, %)			
Medical plan for the economically disadvantaged	14 (66.7%)	29 (69.0%)	19 (100%)
Mandatory health insurance	6 (28.6%)	11 (26.2%)	0 (0.00%)
Comorbidities (n, %)			
Diabetes	10 (47.6%)	13 (31.0%)	2 (11.1%)
Cardiovascular diseases	13 (61.9%)	31 (75.6%)	14 (82.4%)
High blood pressure	14 (66.7%)	28 (66.7%)	13 (72.2%)
Initial nephropathy (n, %)			
Diabetic nephropathy	8 (38.1%)	11 (25.6%)	0 (0.00%)
Glomerulonephritis	5 (23.8%)	8 (18.6%)	7 (36.8%)
Hypertensive nephropathy/ vascular nephropathy	5 (22.8%)	9 (20.9 %)	7 (36.9%)
Tubulo-interstitial nephropathy/autosomal dominant polycystic kidney disease/ obstructive nephropathy/ others	3 (14.3%)	15 (34.9%)	5 (26.3%)
Disease duration (months) (median [IQR])	52.0 [30.0; 84.0]	43.0 [22.5; 79.5]	48.0 [27.0; 65.0]
eGFR (mL/min/1.73m^2^) (median [IQR])	45.0 [37.0; 49.0]	22.0 [18.5; 24.5]	10.0 [6.00; 12.5]
Hemoglobin (g/dl) (mean ± SD)	12.3 ±1.92	11.4 ± 1.78	9.92 ± 2.17
Serum iron µg/l (median [IQR])	0.56 [0.52; 0.69]	0.70 [0.40; 0.84]	0.61 [0.44; 0.90]
Ferritinemia ng/ml (median [IQR])	74.0 [35.2;121]	75.0 [38.5; 188]	64.5 [37.5; 276]
Antihypertensive treatment (n, %)			
Angiotensin II receptors blockers	6 (28.6%)	13 (30.2%)	5 (26.3%)
ACE inhibitors	7 (33.3%)	10 (23.3%)	5 (26.3%)
Calcic channel inhibitors	3 (14.3%)	14 (32.6%)	14 (73.7%)
Diuretics	7 (33.3%)	20 (46.5%)	7 (36.8%)
Supplementation treatment (n, %)			
Calcium	1 (4.76%)	9 (20.9%)	3 (15.8%)
Vitamin D	3 (23.1%)	4 (17.4%)	1 (12.5%)
Oral iron	2 (9.52%)	10 (23.3%)	11 (57.9%)
Erythropietin	0 (0.00%)	2 (4.65%)	4 (21.1%)

eGFR: estimated glomerular filtration rate; ACE: angiotensin-converting enzyme

**Figure 1:**
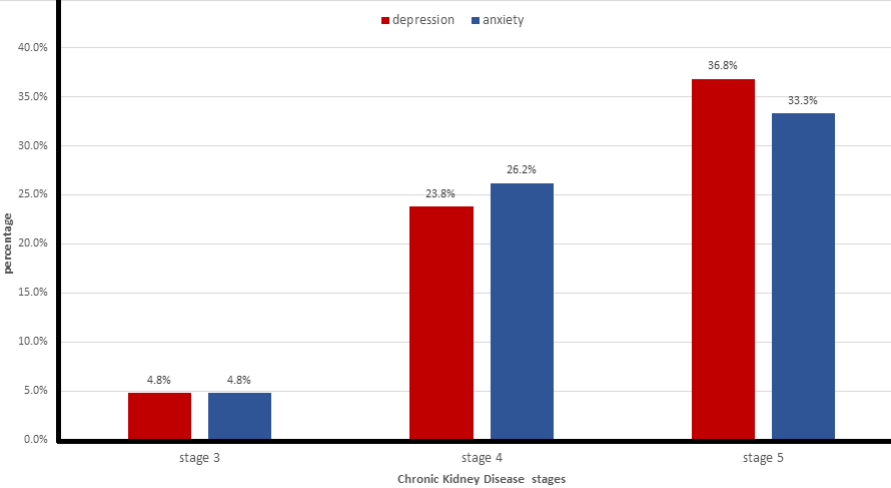
prevalence of depression and anxiety according to chronic kidney disease (CKD) stage

**Depression and anxiety:** the median of the depression sub-score was 5.00 (IQR (2.00; 10.0)), and the median of the anxiety sub-score was 6.00 (IQR (4.00; 9.00)). After using the thresholds, it turns out that 22.0% of patients had probable depression, and 22.2% had probable anxiety. Figure 1 shows the description of the prevalence of anxiety and depression according to the CKD stage. The depression and anxiety sub-scores were significantly different between CKD stages (P=0.008, P=0.004 respectively).

The Spearman´s correlation showed that the eGFR was negatively correlated with both depression (p<0.001; rho= -0.38) and anxiety (p<0.001; rho = -0.4). The simple linear regression (Table 2) showed that depression sub-score was significantly associated with eGFR, serum urea, hemoglobin, and antihypertensive treatment (p<0.001, p=0.008, p=0.01, p=0.03 respectively) and that the anxiety sub-score was significantly associated with diabetes, eGFR and serum urea (p=0.04, p= 0.001, p=0.01 respectively).

**Table 2:** results of simple linear regression evaluating the association between depression/anxiety sub-scores and chronic kidney disease (CKD) patients´ characteristics

Patient’s characteristics	Depression sub-score			Anxiety sub-score		
	**β**	**IC 95%**	**p**	**β**	**IC95%**	**p**
eGFR	-0.14	[-0.21; -0.06]	<0.001	-0.09	[-0.15; -0.03]	0.001
Diabetes	-1.3964	[-3.78; 1]	0.248	-1.83	-3.61; -0.05	0.04
Serum urea	3.071	[0.80; 5.33]	0.008	2.0875	0.39; 3.78	0.0166
Hemoglobin	-0.68	[-1.20; -0.15]	0.01	-0.38	[-0.79; 0.033]	0.07
HBP treatment	1.18	[0.05; 2.31]	0.03	0.15	-0.72; 1.03	0.72

eGFR: estimated glomerular filtration rate; HBP: high blood pressure

The multiple linear regression (Table 3), showed that the decrease in eGFR by one point leads to an increase in depression and anxiety scores by 0.13 points (CI 95% (-0.21; -0.05), P=0.001), and by 0.09 points (CI 95% (-0.15; -0.03), P=0.002) respectively.

**Table 3:** results of multiple linear regression with depression or anxiety sub-scores as dependent variables and eGFR as main independent variable

Sub-scores	B*	CI 95%*	P*	β*	CI 95% *	p*
Depression	-0.14	[-0.21; -0.06]	0.001	-0.11	[-0.23; 0.008]	0.06^a^
				-0.13170	[-0.21 -0.05]	0.001^b^
Anxiety	-0.09	[-0.15; -0.03]	<0.001	-0.05	[-0.15; 0.037]	0.22^a^
				-0.09	[-0.15; -0.03]	0.002^c^

* before adjustment; ^a^ initial model adjusting on: age, sex, eGFR, life condition, hemoglobin, HBP treatment (ARAII, IEC, IC et diuretics), urea, diabetes; ^b^ depression final model eGFR and HBP treatment; ^c^ anxiety final model: eGFR; eGFR: estimated filtration rate; HBP: high blood pressure

## Discussion

**Prevalence of depression and anxiety in CKD:** the current cross-sectional single-center study, showed that one in five patients with CKD presented depression or anxiety disorder before starting renal replacement therapy (RRT). Most authors are of the opinion that mental disease is widespread among CKD patients due to severe physical and psychosocial limitations, which supports our study results [16]. The other studies conducted in our context on this topic targeted only hemodialyzed patients. The first one was conducted in 2003 by Sqalli-Houssaini T *et al*., who found that the prevalence of depression and anxiety among the patients surveyed was 67 and 69.3% respectively [10]. The second one conducted by El Filali A *et al*. [9], in another university hospital, found that 34% of patients had a major depressive episode, 25.2% had anxiety disorder and 16.5% had suicidal ideation [9].

The discrepancies with our study are mainly due to the choice of patients´ category. Knowing that pre-dialysis patients have more autonomy and fewer complications than HD ones the prevalence of depression and anxiety is logically lower in CKD before the start of HD. Also, the tools used to assess depression and anxiety were different.

The prevalence of depression and anxiety among CKD patients has been the subject of several studies around the world but most of them focused on patients undergoing RRT (hemodialysis or peritoneal dialysis). Only a few studies have targeted these psychiatric disorders in the pre-dialysis stages of CKD patients. Thus, the Tasmanian study by McKercher *et al*., which assessed the prevalence of depression and anxiety in 49 patients in stage 4 of CKD, showed that the prevalence of depression and anxiety were 10% and 9% respectively [17]. The study conducted in the Netherlands by Loosman *et al*. [18], on 100 CKD patients with an eGFR ≤ 35 ml/min/1.73 m^2^, found a prevalence of 34% and 31% for depression and anxiety respectively among recruited patients. In Taiwan, the study by Tu CY *et al*. on 326 patients with CKD (stages 1 to 5) not dialyzed, found a very low prevalence of depression and anxiety (3.1% each) among patients, but the health-related anxiety was higher (18%) [19]. The Turkish study conducted on 120 patients with CKD not yet on dialysis found that the prevalence of anxiety and depression were 53.4% and 35% respectively [20]. The Indian cross-sectional study on 200 patients of CKD stages 3 to 5, found that the prevalence of anxiety, depression, and insomnia were found to be 71%, 69%, and 86.5% respectively. The review article by McKercher *et al*., reported that around 22% of individuals with pre-dialysis CKD fulfill the criteria for major depression while 37-55% report depressive symptoms, and around 28% of patients with CKD 3-5 reported high levels of anxiety symptoms [21]. The discrepancies in prevalence between these studies and ours, may be due to the small sample size [17] or higher sample size [4,18], the restriction to just one stage of CKD [17], or the use of different tools to assess psychiatric disorders [18,19].

Some studies focused only on depression without studying anxiety, like the systematic review by Palmer *et al*. which showed that the estimated prevalence of depression varied by stage of CKD and the tools used for diagnosis. They found out that the prevalence of interview-based depression estimates was somewhat less precise for CKD stages 1-5 (21.4% (CI, 11.1-37.2)). This systematic review suggested that self-report scales may overestimate the presence of depression, particularly in the dialysis setting [6].

Overall, previous studies established depression as the primary mental health problem of patients with CKD. The prevalence of depression among patients who have CKD is estimated to be between 20% and 30% [22,23], which corresponds to the results of this study: 22% of the patients in this study had depression. On the other hand, it has been demonstrated that there are increasing levels of anxiety among patients with CKD. A previous study estimated the prevalence rate of anxiety in patients with CKD to be 12% to 52% [23,24]; which also corresponds to the results of the current study (22.2% of patients had anxiety).

**Factors associated with depression and anxiety in CKD:** in the current study, depression, and anxiety in CKD patients were both significantly associated with eGFR before and after adjustment on several confounding factors, that depression sub-score was significantly associated with eGFR, serum urea, hemoglobin, and antihypertensive treatment and that the anxiety sub-score was significantly associated with diabetes, eGFR and serum urea. Our results partially concord with Aggarwal *et al*., which found that anxiety, and depression scores have a strong negative correlation with eGFR, hemoglobin, serum calcium (p <0.01) and a positive correlation with total leukocyte count, blood urea, serum creatinine, and serum phosphate (p <0.05) [4].

Our findings discorded with several studies because of the absence of comparison of the prevalence of depression and anxiety according to the eGFR or the CKD stage, or because of the enrolment of just one category of patients [24]. Thus, the Turkish study showed that both depression and anxiety were related to gender and age (p <0.05) [20]. The study by Tu CY *et al*. showed that depression was significantly associated with age (p<0.01), and exercise (p=0.003), the anxiety was related to high blood pressure (p=0.02) and health-related anxiety was associated with age (p<0.001), exercise (p=0.01) and alcohol consuming (p=0.005) [19]. The Dutch study by Loosman *et al*. showed that anxiety was associated with the female gender (p=0.04) and depression was associated with cardiovascular diseases (p=0.02) [18]. Hedayati *et al*. study found that major depressive episodes did not vary significantly among different CKD stages, but it was associated with diabetes mellitus, comorbid psychiatric illness, and a history of drug or alcohol abuse [24].

In the local literature, the study by Sqalli *et al*., depression has been shown to be associated with several hemodialysis adequacy markers like high blood pressure, interdialytic weight intake, nutritional parameters like serum albumin concentration, and serum creatinine concentration. Depression was more frequent in women, diabetics, and patients with C hepatitis [10]. The study by El Filali *et al*., revealed an association between, major depressive episodes and three factors: living alone, the presence of pain, and anxiety disorders [9], and anxiety showed that anxiety was associated to the age and quality of life [9]. The discrepancies with our study are mainly due to the difference in patients´ categories (hemodialyzed vs non-dialyzed).

**Study´s strengths and limitation:** at our knowledge, this is the first study in a context like ours on psychiatric disorders in CKD patients before the start of renal replacement therapy. The study was conducted in a university hospital that receive population from all around the region. Despite its strengths, this study has several limitations: the first was that the study participants were included from a single center which makes its representability doubtful. The second: we only assessed depressive and anxiety symptoms at baseline, whereas depressive and anxiety symptoms may change over time [18]. The third: was the size of the sample. Also, further, depression and anxiety were diagnosed using a clinically administered scale rather than standardized clinical interviews which may overestimate the prevalence of these disorders [17]. However, the used tool measures in the current study have been validated in renal populations and are brief and easy to administer and interpret [17].

## Conclusion

This study showed that depression and anxiety are strongly related to the CKD progression, which should motivate both doctors and nurses to improve their psychological care toward CKD patients. Also, future research should be conducted to study the evolution of depression and anxiety symptoms in CKD patients using clinical interviews and validated scales.

### What is known about this topic

- *Mental disorders are widespread among CKD patients due to severe physical and psychosocial limitations, which supports our study results;*
- *In Morocco, the prevalence of depression in hemodialysis patients ranged between 34% and 67% and the prevalence of anxiety ranged between 25.2% and 69.3%*.

### What this study adds

- *To our knowledge, this is the first study targeting mental disorders in CKD before the start of RRT conducted in a country like ours;*
- *Depression and anxiety affect one in five patients suffering from CKD patients before RRT;*
- *Both depression and anxiety are negatively correlated to eGRF even after adjustment on several confounding factors*.
